# Assessment of nutritional status in chronically dialyzed patients: high prevalence of malnutrition based on subjective global assessment, simplified nutritional appetite questionnaire, anthropometry and serum albumin analysis – a cross-sectional study

**DOI:** 10.1080/07853890.2025.2578731

**Published:** 2025-10-29

**Authors:** Agnieszka Pluta, Justyna Przybyszewska, Paweł Stróżecki, Mariusz Flisiński, Rafał Donderski

**Affiliations:** ^a^ Division of Community Nursing, Faculty of Health Sciences, Ludwik Rydygier Collegium Medicum in Bydgoszcz of the Nicolaus Copernicus University in Toruń, Toruń, Poland; ^b^ Department of Nutrition and Dietetics, Collegium Medicum in Bydgoszcz, Nicolaus Copernicus University in Torun, Toruń, Poland; ^c^ Department of Nephrology, Hypertension and Internal Diseases, Faculty of Medicine, Ludwik Rydygier Collegium Medicum in Bydgoszcz of the Nicolaus Copernicus University in Toruń, Toruń, Poland

**Keywords:** Malnutrition, dialysis, chronic kidney disease, appetite

## Abstract

**Introduction:**

Malnutrition is common in dialysis patients and is associated with increased morbidity and mortality. Body Mass Index (BMI) may underestimate nutritional disturbances; therefore, multidimensional assessment is recommended. This study evaluated nutritional status, appetite and risk of weight loss in chronically dialyzed patients, and compared different methods, Subjective Global Assessment (SGA), Simplified Nutritional Appetite Questionnaire (SNAQ), anthropometry and biochemical markers.

**Methods:**

A total of 105 chronically dialyzed patients were included (88.6% on hemodialysis, 11.4% on peritoneal dialysis). Nutritional status was assessed using SGA (seven-point scale), SNAQ, anthropometry (BMI, mid-arm circumference), and serum concentrations of albumin and C-reactive protein (CRP) as the markers of nutritional and inflammatory status. The Shapiro–Wilk test was used for normality; group comparisons employed Student’s *t*-test or Mann–Whitney U test and Kruskal–Wallis ANOVA for multiple groups. Correlations were analyzed using Spearman’s coefficient.

**Results:**

Malnutrition, according to SGA (≤5 points), was present in 54.3% of patients, whereas BMI <20 kg/m^2^ was observed in only 9.8%. Among those with low BMI, 90% were malnourished by SGA, and 60% had appetite impairment. Appetite disorders (SNAQ ≤14 points) occurred in 42% of patients and were associated with lower albumin levels, smaller mid-arm circumference and lower body weight . BMI positively correlated with SGA, SNAQ and albumin.

**Conclusions:**

Malnutrition is highly prevalent in Polish dialysis patients and is linked with appetite impairment, elevated CRP, reduced albumin and poorer clinical status. BMI alone is insufficient to detect nutritional disturbances. Regular use of multidimensional tools (SGA, SNAQ, albumin) should be employed to enhance the early detection and management of malnutrition in patients with end-stage kidney disease (ESKD).

## Introduction

Chronic kidney disease (CKD) is a condition classified alongside diabetes, hypertension and cardiovascular diseases as one of the twenty-one-century lifestyle diseases. CKD affects approximately 850 million people worldwide, including 47 million in the European Union [1,2]. In Poland, its prevalence is estimated at around 4 million [3]. Patients with end-stage kidney disease (ESKD) require renal replacement therapy [4,5]. Dialysis is one of the treatment options for ESKD, alongside transplantation. The two primary dialysis methods are hemodialysis (HD) and peritoneal dialysis (PD). As of the end of 2022 in Poland, a total of 20,198 patients were undergoing dialysis, including 19,389 with HD and 809 with PD [4]. Cardiovascular diseases remain the leading cause of mortality among the chronically dialyzed patients, while nutritional disorders significantly contribute to increased morbidity and adverse outcomes in this population [4,6].

One of the key factors contributing to increased morbidity and mortality among dialysis patients is protein–energy wasting (PEW), with a reported prevalence ranging from 20% to 70% [7–10]. The risk of malnutrition increases with CKD progression, and patients in stages 4–5 or those undergoing chronic dialysis are at the highest risk of death and PEW-related complications [11]. The development of this condition is multifactorial. It is associated with dietary restrictions (phosphorus, sodium, potassium), chronic inflammation, enhanced catabolism driven by uremic toxins, metabolic acidosis, endocrine disturbances and nutrient loss during dialysis [8–10]. Recent data also emphasize the role of taste disturbances (dysgeusia) and smell disorders (anosmia) in worsening nutritional status among CKD patients [12].

Given the strong association between PEW and increased morbidity and mortality, early detection is crucial in this population [1,8–11,13]. However, despite this knowledge and the recommendations provided by the European Best Practice Guidelines (EBPG), the Kidney Disease Outcomes Quality Initiative (KDOQI) and the Polish Society of Nephrology (PTN) – all of which advocate regular nutritional assessment – malnutrition remains a significant issue among CKD patients [14–17]. The main difficulty lies in the absence of a single ideal marker, as each currently available parameter has its own limitations. A simple indicator such as the Body Mass Index (BMI) often fails to detect the so-called hidden malnutrition. It may be particularly misleading in patients with fluid overload or sarcopenic obesity [16]. Recent studies suggest that multidimensional tools, such as the Subjective Global Assessment (SGA) and the Simplified Nutritional Appetite Questionnaire (SNAQ), which integrate clinical, anthropometric and biochemical parameters, allow for a more comprehensive and sensitive assessment of nutritional status and have greater prognostic value in CKD patients [11,14–16, 19,20]. Nonetheless, evidence is still lacking as to whether different assessment methods provide consistent results in clinical practice and whether they identify the same subgroups of patients at risk of malnutrition.

Therefore, this study aimed to evaluate nutritional status, appetite and risk of weight loss in a cohort of Polish patients with ESKD undergoing chronic dialysis, and to compare the utility of various nutritional assessment methods – beyond BMI – in identifying malnutrition and the risk of its occurrence. In addition, the study sought to highlight discrepancies between the results of the different tools applied.

## Materials and methods

### Participants

The study was conducted in 2022, following approval from the Bioethics Committee of Nicolaus Copernicus University in Toruń (approval number: 343/2022), at the Ludwik Rydygier Collegium Medicum in Bydgoszcz.

The study included 105 chronically dialyzed patients treated at a dialysis center in Bydgoszcz.

Inclusion criteria were age ≥18 years, written informed consent and dialysis duration >6 months. Exclusion criteria were dialysis duration <6 months, lack of consent, signs of infection within the 4 weeks prior to enrollment and chronic inflammatory disease (e.g. rheumatoid arthritis.

To assess the nutritional status of the study participants, a 7-point Subjective Global Assessment (SGA) scale was used. Individual 7-SGA scores were interpreted based on the following criteria:

- 1–3: Severe malnutrition
- 4–5: Moderate malnutrition
- 6–7: Good nutritional status [7,19].

The risk of weight loss and appetite was assessed using the standardized Short Nutritional Assessment Questionnaire (SNAQ). This questionnaire evaluates appetite, satiety, taste perception, and the number of meals consumed daily. The total score in the SNAQ ranged from 4 to 20 points. Scores ≤14 were interpreted as indicative of poor appetite and an increased risk of weight loss within the next 6 months [20].

To assess the energy nutritional status in all enrolled patients, anthropometric measurements were performed, including body weight (kg), height (cm), and mid-upper arm circumference (MUAC, cm). Body weight was measured using a Radwag medical electronic scale with an accuracy of 0.01 kg. Height was measured with a stadiometer (measurement error: 0.1 cm). MUAC was assessed with a measuring tape to the nearest 0.1 cm. In chronically hemodialyzed patients, MUAC was measured on the non-dominant arm, and – if an arteriovenous fistula was present – on the arm contralateral to the fistula. According to the established cut-off points, values <23 cm in men and <22 cm in women were classified as indicative of malnutrition, while values >24 cm in men and >23 cm in women indicated normal nutritional status. All anthropometric measurements were performed once by a staff member of the Department of Human Nutrition and Dietetics to ensure accuracy, consistency and data reliability. Based on height and weight, the body mass index (BMI) was calculated. Normal nutritional status was defined as a BMI of 20.0–24.9 kg/m^2^. Underweight was defined as BMI ≤19.9 kg/m^2^, overweight as BMI 25.0–29.9 kg/m^2^ and obesity as BMI ≥30.0 kg/m^2^ [21]. Three patients did not have BMI calculated due to lower-limb amputation. In hemodialysis patients, anthropometric measurements were obtained after completion of the midweek dialysis session, whereas in peritoneal dialysis patients, they were taken in the morning during routine monthly outpatient visits.

Additionally, to assess protein nutritional status, serum albumin concentrations routinely obtained as part of patient care were used. Normal status was defined as albumin >3.5 g/dL; values of 3.2–3.4 g/dL indicated mild malnutrition, 2.8–3.1 g/dL moderate malnutrition and <2.8 g/dL severe malnutrition [22].

The study also included serum C-reactive protein (CRP) – a marker of inflammation that may affect nutritional parameters – and creatinine, both routinely measured as part of dialysis patient care. CRP concentrations <5 mg/L were considered within the normal range. Levels > = 5 mg/L were regarded as clinically significant inflammation. Albumin, creatinine and CRP determinations were performed at the Department of Laboratory Diagnostics, University Hospital No. 1 in Bydgoszcz. This study was reported in accordance with the Strengthening the Reporting of Observational Studies in Epidemiology (STROBE) guideline.

### Statistical analysis

Statistical inference was performed using the Statistica 13.3 software package (Polish version, TIBCO Software Inc., California, USA). The Shapiro–Wilk test was used to assess the normality of variable distributions. Descriptive statistics were presented as the mean and standard deviation for variables with a normal distribution and as the median and quartiles (Q1, Q3) for variables with a non-normal distribution. Student’s *t*-test or Mann–Whitney U test was used for comparisons between two groups, depending on the nature of the distribution. Differences between more than two groups were analyzed using the Kruskal–Wallis ANOVA test; for categorical variables, the Chi-square test was used. Spearman’s rank correlation coefficient was calculated to assess the relationship between continuous variables with a non-normal distribution. All tests were two-tailed. P values were reported to three decimal places, with *p* < 0.05 considered statistically significant.

## Results

The characteristics of the study population are presented in Table 1. The study included 105 patients, 93 (88.6%) undergoing hemodialysis and 12 (11.4%) undergoing peritoneal dialysis. Men comprised 64.8% of the study population (*n* = 68), while women accounted for 35.2% (*n* = 37). The median age of the patients was 64 (Q1 = 49.0; Q3 = 73.0) years. The average duration of a single hemodialysis session was 4.0 ± 0.3 h. The median dialysis duration since the start of therapy was 103 months. Men had significantly higher height, weight and mid-arm circumference than women (174 cm vs. 160 cm; *p* < 0.001, 79.97 ± 16.9 kg vs. 63.1 ± 12.9 kg; *p* < 0.001, 28.8 ± 3.6 cm vs. 26.3 ± 2.8 cm; *p* = 0.002).

**Table 1.** Characteristics of the study population.

Parameter	Total Group *N* = 105		Men *N* = 68		Women *N* = 37		*p*-value
	Me	Q1; Q3	Me	Q1; Q3	Me	Q1; Q3	
	(x ± SD)	min-max	(x ± SD)	min–max	(x ± SD)	min-max	
**Age [years]**	64.0	49.0; 73.0	65.0	52.0; 72.5	56.0	44.0; 73.0	0.196
**Duration of hemodialysis [weeks]**	103.0	103.0; 106.0	103.0	103.0; 106.0	103.0	103.0; 106.0	0.236
**Creatinine [mg/dl]**	7.9	6.3; 10.1	8.1	6.0; 10.4	7.8	6.5; 9.6	0.771
**Albumin [g/dl]**	3.9	3.7; 4.2	4.0	3.8; 4.2	3.9	3.7; 4.2	0.655
**CRP [mg/l]**	5.1	1.6; 13.0	6.1	2.1; 13.4	4.1	1.6; 12.9	0.280
**Height** **[cm]**	169.0	162.0; 176.0	174.0	167.0; 177.0	160.0	152.0; 166.5	<0.001
**Weight** **[kg]**	(74.2 ± 17.5)	31.2–137.0	(80.0 ± 16.9)	44.8–137.0	(63.1 ± 12.9)	31.2–85.3	<0.001
**BMI [kg/m^2^]**	25.6	22.3; 28.6	25.8	23.1; 29.6	23.6	20.9; 27.6	0.056
**MUAC** **[cm]**	(27.9 ± 3.6)	21.0–36.5	(28.8 ± 3.6)	21.8–36.5	(26.3 ± 2.8)	21.0–32.0	0.002
**SGA**	5.0	4.0; 6.0	5.0	5.0; 6.0	6.0	4.0; 6.0	0.603
**SNAQ**	(15.0 ± 2.7)	6.0–20.0	(15.5 ± 2.6)	9.0-20.0	(14.0 ± 2.7)	6.0–18.0	0.007

Abbreviations are: CRP – C-reactive protein, BMI – Body Mass Index, MUAC – mid-upper arm circumference, SGA – Subjective Global Assessment, SNAQ – Simplified Nutritional Appetite Questionnaire.

The median score for the SGA questionnaire in the overall study population was 5 points. The mean score for the SNAQ questionnaire was 15.0 ± 2.7 points. Men scored significantly higher in the SNAQ questionnaire than women (15.5 ± 2. 6 vs. 14.3 ± 2.7 points; *p* = 0.007).

**Figure 1.**
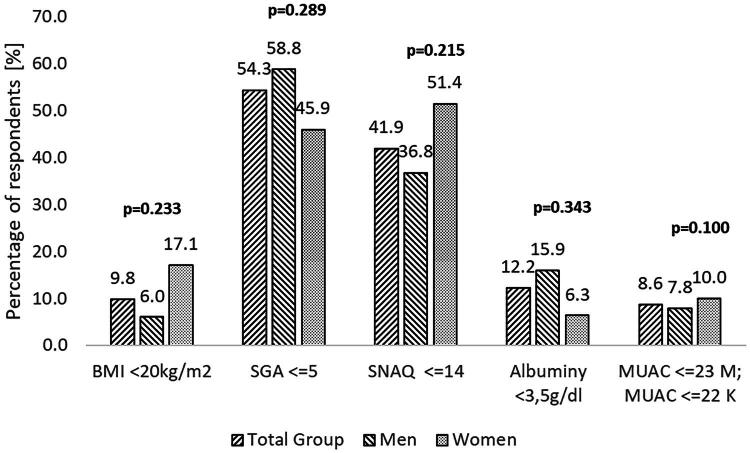
Prevalence of nutritional abnormalities in the study group. Abbreviations are: BMI – Body Mass Index, SGA – Subjective Global Assessment, SNAQ – Simplified Nutritional Appetite Questionnaire, MUAC – Mid-Upper Arm Circumference. *p*-value for the Chi^2^ test.

The nutritional assessment of chronically dialyzed patients conducted in this study revealed abnormalities in both energy and protein nutritional status. Depending on the method applied, nutritional abnormalities were observed in 8.6% to 54.3% of the overall study population, including 6.0% to 58.8% of men and 6.3% to 51.4% of women (Figure 1). A comparative analysis of the results showed no significant differences in the assessed parameters between women and men (Figure 1).

The analysis of the distribution of patients across the interpretation ranges of the 7-SGA scale showed that 45.7% of the patients had a normal nutritional status (SGA ≥6 points). Malnutrition (SGA ≤5 points) was observed in 54.3% of the study participants, with moderate malnutrition (SGA 4–5 points) affecting 47 patients (82.5%) and severe malnutrition affecting 10 patients (17.5%). Due to the small number of patients with severe malnutrition, statistical inference combined the results for this group with those of patients with moderate malnutrition (Table 2).

**Table 2.** Comparison of analyzed parameter values between malnourished patients and those with normal nutritional status.

Parameter	Malnutrition (SGA < =5)*N* = 57		Normal nutritional status (SGA > =6)*N* = 48		*p* value
	Me (x ± SD)	Q1; Q3	Me (x ± SD)	Q1; Q3	
		min-max		min–max	
**Age [years]**	63.0	50.0; 72.0	68.5	48.0; 74.0	0.353
**Duration of Hemodialysis [weeks]**	103.0	103.0-108.5	103.0	103.0; 105.0	0.887
**Albumin [g/dl]**	3.9	3.7; 4.2	4.1	3.8; 4.2	0.249
**CRP [mg/l]**	6.1	1.7; 13.8	4.6	1.6; 11.6	0.246
**BMI [kg/m^2^]**	23.5	21.0; 26.8	27.2	25.0; 29.9	0.001
**MUAC** **[cm]**	(27.4 ± 4.2)	21.0–36.5	(28.5 ± 2.6)	24.0–34.4	0.144
**SGA**	5.0	4.0; 5.0	6.0	6.0; 6.0	<0.001
**SNAQ**	(14.7 ± 3.1)	6.0-20.0	(15.3 ± 2.1)	10.0–19.0	0.233

Abbreviations are: CRP – C-reactive protein, BMI – Body Mass Index, MUAC – mid-upper arm circumference, SGA – Subjective Global Assessment, SNAQ – Simplified Nutritional Appetite Questionnaire.

Patients with a normal nutritional status as assessed by the SGA scale (SGA ≥6 points) demonstrated significantly higher BMI values compared to the group of patients whose SGA scores indicated malnutrition (27.2 kg/m^2^ vs. 23.5 kg/m^2^; *p* = 0.001) (Table 2).

This study did not reveal a statistically significant impact of nutritional status assessed by the SGA scale on the values of other analyzed parameters. However, a significant positive correlation was observed between SGA scores and BMI values (*R* = 0.32; *p* = 0.001).

The mean score obtained on the SNAQ scale for the entire study population was 15.0 ± 2.7. The lowest score recorded was 6, while the highest was 20. Interpretation of individual SNAQ questionnaire results showed that approximately 42% of CKD patients treated with dialysis had impaired appetite (SNAQ ≤14 points) (Figure 1).

It was observed that patients with normal appetite (SNAQ >14 points) had significantly higher albumin levels, BMI and mid-arm circumference compared to patients with impaired appetite (Table 3).

**Table 3.** Comparison of analyzed parameter values between patients with impaired appetite and those with normal appetite.

Parameter	Impaired appetite (SNAQ< =14) *N* = 44		Normal appetite (SNAQ > 14) *N* = 61		*p*-value
	Me (x ± SD)	Q1; Q3	Me (x ± SD)	Q1; Q3	
		min–max		min–max	
**Age [years]**	64.0	46.0; 73.0	64.0	52.0; 73.0	0.673
**Duration of Hemodialysis [weeks]**	103.0	103.0; 106.0	103.0	103.0; 106.0	0.525
**Albumin [g/dl]**	3.8 ± 0.4	2.8–4.4	4.0 ± 0.4	3.1–4.8	0.037
**CRP [mg/l]**	19.2	1.6; 31.3	9.8	1.7; 7.0	0.689
**BMI [kg/m^2^]**	23.4	21.0; 26.5	26.7	23.9; 29.9	0.001
**MUAC** **[cm]**	25.6	23.8; 28.0	29.2 ± 3.2	22.0–36.5	0.001
**SGA**	5.0	4.0; 6.0	5.0	5.0; 6.0	0.288
**SNAQ**	12.5 ± 1.8	6.0–14.0	16.8 ± 1.5	15.0-20.0	<0.001

Abbreviations are: CRP – C-reactive protein, BMI – Body Mass Index, MUAC – mid-upper arm circumference, SGA – Subjective Global Assessment, SNAQ – Simplified Nutritional Appetite Questionnaire.

A statistically significant positive correlation was found between SNAQ scores and BMI values (*R* = 0.32; *p* = 0.001).

The median BMI value for the overall group was 25.6 kg/m^2^. An analysis of individual BMI values revealed that only 32.6% of the studied patients had a normal energy nutritional status (BMI 20–25 kg/m^2^). Among the 102 participants, 41 patients (40.2%) had BMI values indicative of overweight, while 18 patients (17.6%) were classified as obese (BMI ≥30 kg/m^2^). Underweight (BMI ≤19.9 kg/m^2^) was observed in 9.8% of the patients (Table 4).

**Table 4.** Values of analyzed parameters of nutritional status in patients with different BMI.

Parameter	Energy nutritional Status (*N* = 102)								p* value
	I – Malnutrition (BMI ≤19.9 kg/m^2^) *N* = 10		II – Normal (BMI 20–24.9.kg/m^2^) *N* = 33		III – Overweight(BMI 25–29.9 kg/m^2^) *N* = 41		IV – Obesity(BMI ≥30 kg/m^2^) *N* = 18		
	Me	Q1; Q3	Me	Q1; Q3	Me	Q1; Q3	Me	Q1; Q3	
	(x ± SD)	min-max	(x ± SD)	min-max	(x ± SD)	min-max	(x ± SD)	min-max	
**Age [years]**	64.5	44.0;75.0	62.0	45.0; 74.0	67.0	52.0; 73.0	59.0	47.0;71.0	0.442*
**Duration of hemodialysis [weeks]**	102.5	102.0; 103.0	103.0	130.0; 103.0	103.0	103.0; 109.0	105.0	103.0; 109.0	0.001^I-III^ 0.001^I-IV^ 0.006^II-IV^
**Albumin [g/dl]**	3.8	3.7; 3.9	3.9	3.6; 4.1	4.1	3.8; 4.3	4.1	3.9; 4.2	0.093*
**CRP [mg/l]**	5.3	1.6; 31.3	2.6	1.4; 11.0	5.0	2.1; 7.0	8.4	4.6; 17.0	0.567*
**MUAC** **[cm]**	(23.4 ± 1.6)	21.0–25.5	(26.2 ± 2.3)	21.8–31.8	(28.8 ± 2.7)	22.0–34.4	(32.040 ± 2.6)	28.5–36.5	0.001^I-II^ <0.001^I-III^ <0.001^I-IV^ 0.004^II-III^ <0.001^II-IV^ 0.008^III-IV^
**SGA**	4.0	3.0; 5.0	5.0	4.0; 6.0	6.0	5.0; 6.0	6.0	5.0; 6.0	0.045^I-II^ 0.002^I-III^ 0.031^I-IV^ 0.033^II-III^
**SNAQ**	(13.9 ± 3.2)	9.0–18.0	(14.1 ± 2.7)	6.0–18.0	(15.3 ± 2.2)	10.0–20.0	(16.5 ± 2.5)	11.0–20.0	0.023^I-IV^ 0.037^II-III^ 0.003^II-IV^

* *p* value for Kruskal–Wallis Rank ANOVA.

Abbreviations are: CRP – C-reactive protein, BMI – Body Mass Index, MUAC – mid-upper arm circumference, SGA – Subjective Global Assessment, SNAQ – Simplified Nutritional Appetite Questionnaire.

It was demonstrated that the group of 18 patients with BMI values indicative of obesity had a statistically significant longer duration of renal replacement therapy (Me = 105, Q1 = 103.0; Q3 = 109.0). In the group of 10 patients with energy malnutrition, significantly lower scores were recorded in both the SGA (Me = 4, Q1 = 3; Q3 = 5) and SNAQ (x̅=13.9 ± 3.2) questionnaires.

Analysis of MUAC results showed that, in the overall cohort (*N* = 81), low MUAC values indicative of malnutrition were observed in 7 individuals (8.6%), while normal MUAC values were recorded in 74 patients (91.4%). Among men, malnutrition was identified in four patients (7.8%), with the remaining men (92.2%) presenting MUAC values consistent with normal nutritional status. Among women, malnutrition was identified in three patients (10.0%) (Figure 1).

The correlation analysis conducted in this study showed that, in addition to the above-described significant relationships between BMI values and SGA and SNAQ scores, BMI also positively correlated with albumin levels (*R* = 0.31; *p* = 0.004).

The abnormalities in the nutritional status of CKD patients were further highlighted by an analysis of individual albumin concentrations relative to reference values. Protein malnutrition, expressed as low albumin levels, was observed in 11 out of 90 study participants with the measured albumin concentrations. It was found that patients with protein malnutrition (albumin <3.5 g/dL) had significantly higher serum creatinine and CRP levels (Table 5). A statistically significant negative correlation was also observed between albumin and CRP levels (R=–0.217; *p* = 0.041). These results suggest that lower albumin values may, at least in part, be the result of an ongoing inflammatory process and not solely malnutrition. Although the correlation demonstrated in our study was weak (R=–0.217), the data obtained confirm the need to take into account inflammatory markers, such as CRP, when interpreting albumin concentration in the assessment of the nutritional status of dialysis patients.

**Table 5.** Comparison of analyzed parameter values between patients with protein malnutrition and those with normal protein nutritional status.

Parameter	Protein malnutrition (albumin <3.5) *N* = 11		Normal protein nutritional status (albumin > =3.5) *N* = 79		*p* value
	Me (x ± SD)	Q1; Q3	Me (x ± SD)	Q1; Q3	
		min–max		min–max	
**Age [years]**	73.0	61.0; 77.0	64.0	46.0; 73.0	0.153
**Duration of hemodialysis [weeks]**	(103.5 ± 2.0)	101.0–109.0	(105.1 ± 3.3)	101.0–113.0	0.182
**Albumin [g/dl]**	3.2	3.1; 3.3	4.1	3.9; 4.2	<0.001
**CRP [mg/l]**	38.5	4.4; 76.5	5.1	1.6; 12.8	0.010
**BMI [kg/m^2^]**	24.3	21.4; 25.8	25.8	21.9; 29.7	0.225
**MUAC** **[cm]**	(27.3 ± 3.8)	31.8–33.0	(28.2 ± 3.4)	21.0–36.5	0.452
**SGA**	5.0	4.0; 6.0	5.0	4.0; 6.0	0.395
**SNAQ**	(13.8 ± 2.6)	9.0–18.0	(15.1 ± 2.8)	6.0–20.0	0.160

Abbreviations are: CRP – C-reactive protein, BMI – Body Mass Index, MUAC – mid-upper arm circumference, SGA – Subjective Global Assessment, SNAQ – Simplified Nutritional Appetite Questionnaire.

The study analyzed the prevalence of nutritional abnormalities concerning energy nutritional status (Table 6). In the group of 10 patients with energy malnutrition (BMI <20.0 kg/m^2^), impaired appetite and the risk of further weight loss within the next 6 months (SNAQ ≤14) were diagnosed in six patients (60%), malnutrition (SGA ≤5) was observed in nine patients (90.0%) and protein malnutrition (albumin <3.5 g/dL) was present in one patient (10%).

**Table 6.** Prevalence of nutritional abnormalities concerning energy nutritional status assessed by BMI.

Energy nutritional status according to BMI	**SNAQ < =14** N (%)	**SGA < =5** N (%)	**Albumin <3.5** N (%)
**Malnutrition (BMI < 20kg/m^2^) *N* = 10**	6 (60.0%)	9 (90.0%)	1 (10%)
**Normal (BMI > =20 - 25 kg/m^2^) *N* = 33**	20 (60.6%)	23 (69.7%)	5 (15.2%)
**Overweight (BMI > =25 - 30 kg/m^2^) *N* = 41**	12 (29.3%)	16 (39.0%)	4 (9.8%)
**Obesity (BMI > =30kg/m^2^) *N* = 18**	4 (22.2%)	7 (38.9%)	1 (5.6%)
***p*-value**	*p* = 0.009	*p* = 0.003	**

*p*-value for Pearson’s Chi^2^ Test; ** Due to the small sample size of individual subgroups, statistical inference was not performed.

Abbreviations are: SNAQ – Simplified Nutritional Appetite Questionnaire, SGA – Subjective Global Assessment, BMI – Body Mass Index.

In the group of 33 patients with normal energy nutritional status (BMI ≥20–25 kg/m^2^), impaired appetite and the risk of further weight loss were identified in 20 patients (60.6%), malnutrition was observed in 23 patients (69.7%), and protein malnutrition was present in 5 patients (15.2%). In the group of 41 patients with overweight (BMI ≥25–30 kg/m^2^), impaired appetite and the risk of further weight loss were identified in 12 patients (29.3%), malnutrition was observed in 16 patients (39.0%) and protein malnutrition was present in 4 patients (9.8%). Finally, in the group of 18 patients with obesity (BMI ≥30 kg/m^2^), impaired appetite and the risk of further weight loss were identified in 4 patients (22.2%), malnutrition was observed in 7 patients (38.9%) and protein malnutrition was present in 1 patient (5.6%).

## Discussion

Despite two decades of recommendations from EBPG, KDOQI [15–17], and the Polish Society of Nephrology [14], which emphasize the necessity of regular monitoring of the nutritional status of dialysis patients, nutritional abnormalities remain a persistent issue in this group. Malnutrition is commonly observed among dialysis patients and is strongly associated with increased morbidity and mortality [17–20,23]. Considering the clinical importance of malnutrition in this population, early identification is essential to ensure optimal healthcare, improve quality of life and reduce the risk of complications [8–10,24].

Malnutrition in end-stage kidney disease primarily results from uremic toxin burden, increased catabolism driven by chronic inflammation, disease-related dietary restrictions and inadequate nutrition care [8,14, 25,26]. The clinical consequences of malnutrition, including muscle atrophy, particularly of skeletal muscles, lead to physical weakness that significantly limits the exercise tolerance of dialysis patients, thereby severely impairing their quality of life [27]. Moreover, the dialysis process itself, which causes fatigue, adversely impacts the patients’ physical state and their ability to perform daily activities [28]. Studies have shown that post-dialysis fatigue is one of the key factors contributing to reduced adherence to dietary recommendations in over 50% of patients [29].

In this study, the mean SGA score (recommended by EBPG, KDOQI [15–17]) and the Polish Society of Nephrology [14] for the overall group was 5.0, indicating moderate malnutrition. Interpretation of individual SGA scores revealed that malnutrition (SGA ≤5) affected half (54.3%) of the study participants. Among the 54.3% of malnourished patients, moderate malnutrition (SGA 4–5 points) was identified in 47 individuals (82.5%), while severe malnutrition was found in 10 individuals (17.5%). The findings of this study are consistent with those of Borek et al. who reported that malnutrition (SGA ≤5) affected 51.8% of hemodialysis patients at the Dialysis Center of the Clinical University Center in Gdańsk [7].The mean SGA score in their study was slightly higher than in this study, at 5.4. Similar proportions of malnourished dialysis patients were observed in studies conducted in Brazil and Iran [30,31]. In a 2021 Brazilian study of 169 HD patients, malnutrition (SGA ≤5) was observed in 59.8% of patients, with a mean SGA score of 5.2 [30]. Rafinezhad et al. [31] found that 56.4% of 133 HD patients in Iran exhibited moderate malnutrition. Amirkhanloo et al. reported an even higher prevalence (70.7%) in a study of 116 HD patients in Iran, where moderate malnutrition was observed in 69.8% and severe malnutrition in 0.9% of patients [32].

In contrast, studies conducted in Italy [30] and Australia [33] reported nearly half the prevalence of malnutrition among CKD patients on dialysis. In an Italian study of 121 HD patients, 25.6% were identified with malnutrition (SGA ≤5) [30]. The lower prevalence of malnutrition observed by Avesani et al. may be related to the Mediterranean origin of the patients, who typically follow an anti-inflammatory diet rich in spices with anti-inflammatory properties and monounsaturated fatty acids. The patients in this study also exhibited low predialysis hsCRP protein levels (1.41 mg/L; 0.34–3.84 mg/L) [30]. In contrast, the median CRP level in this study was 5.14 mg/L, which was three times higher than the levels observed by Avesani et al. [30].

An even lower prevalence of malnutrition (20%) was reported by Desbrow et al. in a study of 60 HD patients treated at a private hospital in Australia [33]. The authors suggested that the low prevalence of malnutrition in their study population may be due to the high socioeconomic status of the participants, which allowed them to afford treatment in a private clinic [33].

The nutritional abnormalities observed in CKD patients treated with dialysis in this study were also highlighted by an analysis of individual Short Nutritional Assessment Questionnaire (SNAQ) scores. It was noted that nearly 42% of patients had SNAQ scores indicating impaired appetite and a risk of weight loss within the next 6 months (SNAQ ≤14 points). Appetite disturbances were observed in approximately 37% of men and 51% of women. Similar findings were reported by Borek et al. who noted that appetite disturbances (SNAQ ≤14 points) were present in 40% of HD patients (35 out of 86 patients) [7]. The results of this study are also consistent with those obtained in a group of 233 Japanese HD patients over 65 years of age [34]. In this study, 49.8% of patients had SNAQ scores indicating appetite disturbances and a risk of weight loss within 6 months (SNAQ ≤14 points), with disturbances noted in 42.8% of men and 59% of women [34]. Appetite disturbances have a significant and complex impact on patients’ nutritional status. Loss of appetite can lead to malnutrition, particularly in dialysis patients, where the challenges associated with the therapeutic process (e.g. post-dialysis fatigue) can further reduce their desire to eat [35].

The latest data also emphasize the importance of smell (anosmia) and taste (dysgeusia) disorders in the deterioration of nutritional status in patients with CKD. A systematic review by Ferrara et al. showed a link between anosmia and poorer nutritional status [12]. In contrast, the NHANES population studies showed that patients with CKD had a significantly higher prevalence of smell disorders (30% vs. 15% in the general population) [36]. In the cited study by Chewcharat et al., a significantly higher risk of smell disorders in patients with CKD (OR = 1.47; *p* = 0.02) and an association between anosmia and reduced muscle strength are observed, which could be a consequence of protein malnutrition (OR = 1.72 for every 10 kg decrease in grip strength; *p* = 0.005) [36]. In our own study, loss of appetite, assessed on the basis of an SNAQ score ≤ 14 points, affected 41.9% of patients overall, with a higher prevalence in women (51.0%) than in men (37.0%). Similar results were obtained by Fitzgerald et al. who showed that 43.8% of hemodialysis patients had taste abnormalities [37]. A similar percentage was also reported by Dawson et al. indicating that 38% of patients with CKD reported taste changes, significantly associated with upper gastrointestinal symptoms and malnutrition [38]. An even higher percentage – 53% of patients with CKD reporting taste dysfunction – was observed by Konstantinova et al. [39]. However, the authors pointed out that their results may be overestimated, as they were based solely on patients’ subjective responses regarding taste changes. A comparison of the data obtained with the results of other authors indicates that the percentage of patients with loss of appetite in our study is within the range observed in other populations with chronic kidney disease, which further confirms the important role of taste and appetite disorders in the development of malnutrition in dialysis patients. The main pathogenetic factors of olfactory and taste function impairment are: uremic toxins, inflammation and dialysis-related disorders [40,41]. It has been reported that in patients after kidney transplantation, partial remission of dysgeusia and anosmia is possible [40,41]. The observations made by the above-mentioned authors indicate that the assessment and monitoring of taste and smell disorders should be an important part of the overall diagnosis of malnutrition in CKD, as they can lead to a decrease in appetite, food aversion and, secondarily, to the development of nutritional deficiencies and deterioration of clinical condition.

The nutritional abnormalities observed in this study were also evident in the analysis of individual albumin levels. Approximately 12.2% of patients exhibited protein malnutrition (albumin levels <3.5 g/dL), while the remaining 87.8% had albumin levels above 3.5 g/dL, indicating normal protein nutritional status. The median albumin level in the overall group was 3.92 g/dL.

The average albumin level calculated in this study is comparable to results obtained by other authors [30,33]. The mean albumin level in Italian HD patients was 3.60 g/dL [30], while in Brazilian HD patients, it was 3.89 g/dL [30]. Similar albumin levels were observed in HD patients in Australia (3.8 g/dL) [31] and Egypt (3.4 g/dL) [42].

BMI is widely used to evaluate the energy nutritional status of dialysis patients. In this study, the median BMI was 25.55 kg/m^2^. In other studies available in the scientific literature, BMI values among HD patients were similar, ranging from 21.4 kg/m^2^ to 27 ± 7 kg/m^2^ [30,43–45]. In this study, BMI values indicative of underweight (BMI ≤19.9 kg/m^2^) were observed in 9.8% of patients, including 6.0% of men and 17.1% of women. These results are consistent with findings in a group of 195 HD patients in Pakistan, where 10.3% of patients had BMI values indicating underweight [44]. Similarly, in a study analyzing retrospective data from the Korean Society of Nephrology, 15.1% of patients out of 10,327 HD patients exhibited energy malnutrition [45].

Borek et al. reported BMI values indicative of underweight in 16% of HD patients in the Gdańsk Dialysis Center [7]. In contrast, a study of 123,383 HD patients in the United States found that underweight (BMI <18.5 kg/m^2^) was significant for 5% of the population [43].

In this study, only 32.3% of patients had BMI values within the reference range (20–24.9 kg/m^2^). In other studies, normal energy nutritional status (BMI 18.5–24.9 kg/m^2^) was observed in 41% to 73.4% of HD patients [7,43–45]. Overweight, as indicated by a BMI of 25.0–29.9 kg/m^2^, was observed in 40.2% of patients in this study, while obesity (BMI ≥30.0 kg/m^2^) was found in 17.6%.

Other scientific studies reported a lower prevalence of overweight among HD patients, ranging from 6.6% in Korean dialysis patients [45] to 24.6% in Pakistani patients [44]. In the United States, overweight was significant in nearly one-third of HD patients (29%) [43]. Among patients in the Gdańsk Dialysis Center, 19% were classified as overweight [7]. Obesity was reported in 0.78% of Korean HD patients, 9.7% of patients in Pakistan [44] and 11% of patients in the Gdańsk study [7]. In the US, obesity was observed in 25% of HD patients [43].

The observed differences in the prevalence of overweight and obesity among dialysis patients may be related to factors such as the age of the study population, duration of dialysis, comorbidities, cultural differences and the socio-economic status of individual populations.

It should also be noted that assessing nutritional status is a complex process, and it poses significant challenges due to the lack of a perfect diagnostic tool without limitations. These findings confirm that BMI alone is not a reliable tool for assessing nutritional status in older adults receiving dialysis, as it does not account for muscle mass, hydration status or the distribution of adipose tissue. This is particularly problematic in elderly individuals, where sarcopenia and functional decline may occur despite normal or elevated BMI values. Therefore, comprehensive tools such as SGA or SNAQ are essential to identify malnutrition early and initiate appropriate interventions to maintain physical function and quality of life. In this study, among 55 patients diagnosed with malnutrition (SGA ≤5), only 10 had BMI values indicating underweight (BMI <20 kg/m^2^). For the remaining 45 patients, BMI values did not indicate energy malnutrition and fell within the following ranges: BMI ≥20–25 kg/m^2^ for 33 patients, BMI ≥25–30 kg/m^2^ for 16 patients and BMI ≥30 kg/m^2^ for the remaining 7 patients.

This confirms previous observations that BMI is not a reliable tool for diagnosing malnutrition, especially among HD patients. BMI is simple, non-invasive and inexpensive, but it does not account for muscle, fat and bone composition in the body, nor does it consider the distribution of fat or hydration status, which is particularly crucial for dialysis patients [46–48].

## Limitations

This study has several important limitations that should be taken into account when interpreting the results. First, its cross-sectional nature prevents the assessment of cause-and-effect relationships between malnutrition and the analyzed clinical and biochemical indicators. The lack of long-term observation also makes it impossible to determine the extent to which the results obtained can predict significant clinical events such as hospitalizations, complications or mortality. Second, the inclusion of patients from a single facility may limit the possibility of generalizing the results to the wider population of dialysis patients in Poland. Finally, the small size of some subgroups (e.g. patients with severe malnutrition) limits the statistical power of the analyses. Despite these limitations, our study highlights the importance of multidimensional assessment of nutritional status in patients with ESRD and may serve as a starting point for further prospective studies, which are planned.

## Summary

Malnutrition is a common issue among dialysis patients, often of moderate or severe intensity, highlighting the need for continuous monitoring of their nutritional status. Impaired appetite, observed in a significant proportion of patients, may be one of the contributing factors to nutritional abnormalities.

The findings of this study suggest that chronically dialyzed patients require regular and comprehensive nutritional assessments to identify and counteract malnutrition and its consequences.

## Data Availability

The data presented in this study are available on request from the corresponding author. The data are not publicly available to protect the study participants from potential identification based on their personal information provided in the study variables.
